# Health Care Utilization With Telemedicine and In-Person Visits in Pediatric Primary Care

**DOI:** 10.1001/jamahealthforum.2024.4156

**Published:** 2024-11-22

**Authors:** Scott D. Casey, Jie Huang, Daniel D. Parry, Tracy A. Lieu, Mary E. Reed

**Affiliations:** 1 Kaiser Permanente Division of Research, Pleasanton, California; 2 The Kaiser Permanente CREST Network, Pleasanton, California; 3 The Permanente Medical Group, Vallejo, California; 4 The Permanente Medical Group, Daly City, California; 5 The Permanente Medical Group, Oakland, California

## Abstract

**Importance:**

Telemedicine is an increasingly used yet understudied vehicle to deliver pediatric primary care. Evidence detailing downstream health care utilization after telemedicine visits is needed.

**Objective:**

To compare pediatric primary care conducted via telemedicine (video or telephone) with in-person office visits with regard to physician medication prescribing and imaging and laboratory ordering and downstream follow-up office visits, emergency department (ED) visits, and hospitalizations.

**Design, Setting, and Participants:**

This cohort study included all patients younger than 18 years who had scheduled primary care appointments with a pediatrician from January 1 to December 31, 2022, in the Kaiser Permanente Northern California health system, a large integrated health care delivery system offering in-person office visits, video visits, or telephone visits for pediatric primary care.

**Exposure:**

Pediatric primary care in-person visit, telephone visit, or video visit.

**Main Outcome and Measures:**

Rates of physician medication prescribing and imaging and laboratory ordering during an index telemedicine or office visit and rates of in-person office visits, ED visits, and hospitalizations within 7 days after the visit, adjusted for patient and clinical characteristics.

**Results:**

Of 782 596 total appointments (51.1% male) among 438 638 patients, telemedicine was used for 332 153 visits (42.4%). After adjustment, there was more medication prescribing for in-person visits (39.8%) compared with video visits (29.5%; adjusted difference, −10.3%; 95% CI, −10.6% to −10.0%) or telephone visits (27.3%; adjusted difference, −12.5%; 95% CI, −12.5% to −12.7%). There was also more laboratory ordering for in-person visits (24.6%) compared with video visits (7.8%; adjusted difference, −16.8%; 95% CI, −17.0% to −16.6%) or telephone visits (8.5%; adjusted difference, −16.2%; 95% CI, −16.3% to −16.0%). There was more imaging ordering for in-person visits (8.5%) compared with video visits (4.0%; adjusted difference, −4.5%; 95% CI, −4.6% to −4.4%) and telephone visits (3.5%; adjusted difference, −5.0%; 95% CI, −5.1% to −4.9%). After adjustment, fewer in-person follow up visits occurred for index visits that were in-person (4.3%) compared with video (14.4%; adjusted difference, 10.1%; 95% CI, 9.9%-10.3%) or telephone (15.1%; adjusted difference, 10.8%; 95% CI, 10.7%-11.0%) visits. The rate of ED visits following an in-person visit was slightly lower (1.75%) compared with after video visits (2.04%; adjusted difference, 0.29%; 95% CI, 0.21%-0.38%) or telephone visits (2.00%; adjusted difference, 0.25%; 95% CI, 0.18%-0.33%). There was no statistically significant difference in the 7-day rate of hospitalizations.

**Conclusions and Relevance:**

In this cohort study, telephone and video visits for pediatric primary care were associated with less prescribing and ordering than in-person visits. Telemedicine visits were associated with modestly higher rates of subsequent in-person visits and slightly higher rates of ED visits, and there was no difference in hospitalizations. Telemedicine appears to be a useful vehicle for health care delivery in the pediatric population, although it is not a universal substitute for in-person visits.

## Key Points

**Question:**

Are pediatric primary care telephone or video visits associated with more medication prescribing, imaging and laboratory ordering, in-person follow-up visits, emergency department (ED) visits, or hospitalizations compared with in-person visits?

**Findings:**

In this cohort study of 782 596 appointments among 438 638 patients, compared with in-person visits, telemedicine visits were associated with less prescribing and ordering, modestly higher rates of in-person visits, and slightly more ED visits. There was no significant difference in hospitalizations.

**Meaning:**

The findings suggest that primary health care delivery that uses telemedicine appears to be meeting patients’ pediatric primary care needs, although it is not a universal substitute for in-person visits.

## Teaser

This cohort study compares rates of physician prescribing and ordering and downstream health care utilization associated with pediatric primary care telemedicine visits vs in-person visits.

## Introduction

Telemedicine emerged as a popular vehicle for health care delivery during the COVID-19 pandemic despite few large studies demonstrating its ability to meet the needs of more than 12 million US children now using it annually.^1,2,3,4,5^ As we enter a postpandemic era, it is important to understand how telemedicine compares with traditional in-person care to guide resource allocation in the pediatric outpatient setting and to support national policy decisions to preserve ongoing pediatric telemedicine access.^6,7^

Previous studies have demonstrated that telemedicine is acceptable to patients and physicians to provide outpatient pediatric care.^8,9,10^ However, few studies have focused on telemedicine’s effectiveness in substituting for in-person pediatric visits or its associated downstream emergency department (ED) and hospital utilization. The results of a few small studies have found no significant association between telemedicine and increases in downstream health care utilization, but these results require confirmation in a larger population.^8,11^

The Kaiser Permanente Northern California (KPNC) telemedicine options include telephone visits and video visits. Previous work from our group demonstrated lower rates of medication prescribing and imaging orders^12^ and slightly higher subsequent in-person visits associated with telemedicine primary care visits compared with in-person primary care visits in an adult population.^13^ However, the effect of telemedicine on health care utilization in the pediatric population is unknown. Further evidence is needed regarding the efficacy of pediatric primary care telemedicine by quantifying differences in the need for downstream follow-up care.^7,14^ In the current study, we compared medication prescribing and imaging and laboratory ordering during an in-person office or telemedicine visit and health care utilization within 7 days after the visit.

## Methods

### Setting

This cohort study examined primary care pediatric visits in a large, integrated health care delivery system, KPNC, which includes nearly 4.5 million members whose demographic characteristics are reflective of the regional population.^15^ KPNC uses a comprehensive electronic health record (EHR) that includes outpatient, emergency, inpatient, laboratory, imaging, and pharmacy history. The EHR also offers a patient portal where patients can self-schedule office or telemedicine pediatric visits. Since 2016, KPNC members have had the choice of telephone, video, or in-person primary care visits. Primary care visits are scheduled based on patients’ preferences and are not directive. The capitated system does not bill patients or insurers for telemedicine visits. The institutional review board of the Kaiser Foundation Research Institute approved the study protocol and materials. The institutional review board granted a waiver of informed consent because this data-only study was determined to be minimal risk. This cohort study followed the Strengthening the Reporting of Observational Studies in Epidemiology (STROBE) reporting guideline.^16^

### Study Population

We studied all completed primary care pediatrics appointments from January 1 through December 31, 2022, including only index visits (1) with a chief concern other than a routine well-child visit and (2) without any other clinical visits within the 7 days prior to define a relatively distinct patient-initiated, care-seeking episode. The health system recommended in-person visits for routine pediatric health care (ie, vaccination appointments, well-child appointments) and telemedicine visits for SARS-CoV-2 infection–associated concerns. For this reason, these visit types were excluded from the study population. All study data were obtained using the EHR and other automated data sources.

### Outcome Measures

For each study index visit, we identified any medication prescribing, laboratory orders, and imaging orders associated with the visit, including a subset of prescriptions specifically for antibiotics. We grouped the clinical concern of visits using the diagnosis grouping system of the *International Statistical Classification of Diseases and Related Health Problems, Tenth Revision (ICD-10)* for pediatric diagnoses in EDs.^17^ To characterize short-term follow-up health care utilization, we extracted all primary care office visits with a pediatrician, ED visits, and hospitalizations within 7 days after the index primary care visit, including same-day visits. We examined each outcome in the full sample of all patient visits as well as stratified by area of clinical concern.

### Covariates

We compared outcomes associated with index visit type, accounting for covariates with literature precedence for an association with visit-type choice,^13,18,19^ including patient sociodemographic characteristics (age, sex, race and ethnicity, neighborhood socioeconomic status, and language), technology access in the prior year (neighborhood internet access, mobile portal access), clinic visit barriers (driving distance from home to primary care facility, paid facility parking), video visit experience (any in prior year), if the visit was with the patient’s usual primary care practitioner or another in the same practice, clinical comorbidities and health care utilization history (Elixhauser score 0 or ≥1, any ED visit in the prior year, or any hospitalization in the prior year), and context of the studied index visit, including appointment booking day (Monday through Thursday, Friday, or Saturday or Sunday), visit day (Monday through Thursday or Friday through Sunday), visit time (morning or afternoon), days between appointment booking and visit (same day, 1 day, 2-7 days, or ≥8 days), *ICD-10* grouping, medical center, and calendar month. We used the patient’s residential address from the EHR to define patient neighborhood socioeconomic status (2010 US census measures at the census block group level) and neighborhood residential high-speed internet access level (Federal Communications Commission census tract–level data) (eTable in Supplement 1). Race and ethnicity were ascertained by self-report. Categories were Asian, Black, Hispanic, White, and other (American Indian or Alaska Native, Hawaiian or Pacific Islander, and unknown).

### Statistical Analysis

We used multivariable logistic regression to examine associations between index visit type (in-person, telephone, or video) and outcomes (medication prescribing and/or imaging or laboratory ordering at the index visit and 7-day in-person visits, ED visits, or hospitalizations), with adjustment for all aforementioned covariates. We examined each outcome using a separate logistic regression model since each outcome represents a clinically distinct action and outcome. Standard errors were adjusted for repeated visits by the same patient by clustering observations by patient with a robust variance estimator. For easier interpretation, we calculated an adjusted rate for each outcome from the multivariable logistic regression and adjusted difference between telephone or video visits and office visits using marginal standardization (using the margins postestimation command in Stata, version 17.0 [StataCorp LLC]). All analyses were conducted using 2-sided tests for significance and *P* < .05 as the threshold for significance, in Stata, version 17.0.

## Results

**Figure 1.**
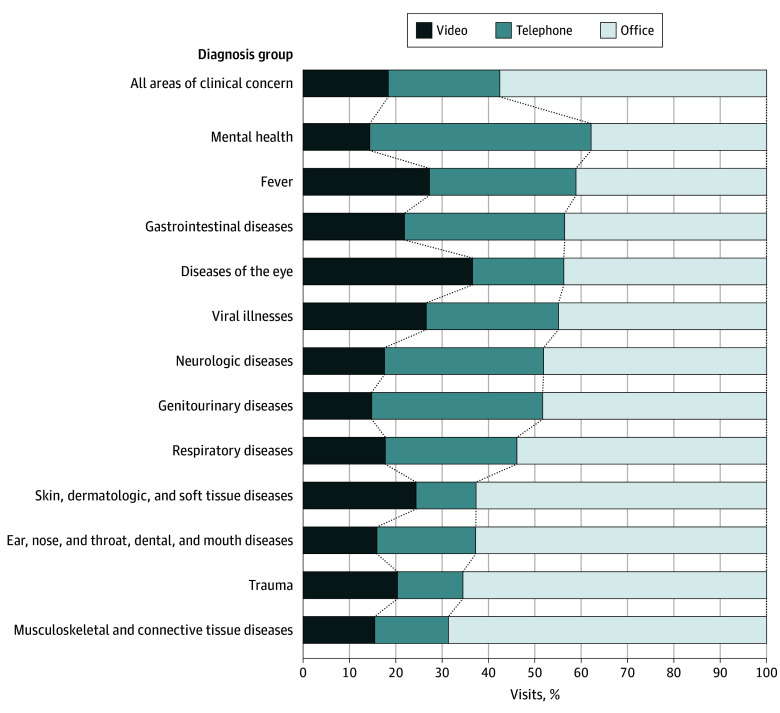
Visit Modality by Clinical Areas Percentages are among all visits in each diagnosis group.

Of 782 596 primary care visits scheduled by 438 638 patients, 450 443 (57.6%) were in-person office visits and 332 153 (42.4%) were telemedicine visits (143 960 video visits [18.4%] and 188 193 telephone visits [24.0%]). Overall, 25.3% of visits were for patients younger than 2 years; 48.9%, for female patients, and 51.1%, for male patients. A total of 19.6% of visits were for Asian individuals; 6.5%, for Black individuals; 29.2%, for Hispanic individuals; 33.7%, for White individuals; and 11.0%, for individuals with other race. Of the total visits, 93.4% were for English-speaking individuals and 21.3% for those who resided in neighborhoods with low socioeconomic status. Approximately half of all visits (51.8% overall, 53.0% in-person, 53.1% telephone, and 46.0% video) were with the patient’s usual pediatrician (Table). Telemedicine and in-person visits were used differentially based on area of clinical concern. For example, telephone and video visits delivered most visits for mental health and were used the least for musculoskeletal and connective tissue disorders (Figure 1).

**Table.** Patient Characteristics

Characteristic	Visits, %			
	All (N = 782 596)	Office (n = 450 443)	Video (n = 143 960)	Telephone (n = 188 193)
Age group, y				
<1	11.06	13.31	9.86	6.61
1-2	14.27	13.72	17.00	13.49
3-5	18.72	17.98	19.64	19.80
6-9	19.07	18.24	18.85	21.22
10-13	17.00	16.82	16.07	18.15
14-17	19.88	19.93	18.58	20.74
Sex				
Female	48.86	48.74	48.87	49.15
Male	51.14	51.26	51.13	50.85
Race and ethnicity				
Asian	19.63	18.97	23.79	18.01
Black	6.50	5.88	6.42	8.05
Hispanic	29.23	28.36	27.65	32.52
White	33.67	33.41	33.61	34.36
Other^a^	10.97	13.38	8.52	7.06
Low neighborhood SES^b^	21.34	20.68	20.54	23.55
Low neighborhood internet access^c^	27.87	27.22	26.88	30.20
English speaking	93.43	92.74	95.66	93.37
Visit with usual PCP	51.76	53.03	46.03	53.11
Video visit in prior year	42.19	38.50	52.35	43.26
Driving distance, min				
≤10	41.20	42.04	39.62	40.40
11 to <20	40.46	40.32	40.94	40.45
≥20	17.06	15.80	18.90	18.69
Elixhauser score ≥1	9.22	10.37	6.63	8.46

Abbreviations: PCP, primary care physician; SES, socioeconomic status.

^a^ Other included American Indian or Alaska Native, Hawaiian or Pacific Islander, and unknown.

^b^ Defined as at least 20% of households with incomes below the federal poverty level or at least 25% of residents 25 years or older with less than a high school education in the census block group.

^c^ Defined as less than 80% of households with a residential fixed high-speed connection with at least 10 Mbps downstream and at least 1 Mbps upstream in the census tract based on US Federal Communications Commission data.

After adjustment, there was more medication prescribing for in-person visits (39.8%) compared with video visits (29.5%; adjusted difference, −10.3%; 95% CI, −10.6% to −10.0%) and telephone visits (27.3%; adjusted difference, −12.5%; 95% CI, −12.5% to −12.7%) (eFigure 1 in Supplement 1) and more laboratory ordering for in-person visits (24.6%) compared with video visits (7.8%; adjusted difference, −16.8%; 95% CI, −17.0% to −16.6%) and telephone visits (8.5%; adjusted difference, −16.2%; 95% CI, −16.3% to −16.0%) (eFigure 2 in Supplement 1). Similarly, imaging ordering was higher for in-person visits (8.5%) compared with video visits (4.0%; adjusted difference, −4.5%; 95% CI, −4.6% to −4.4%) and telephone visits (3.5%; adjusted difference, −5.0%; 95% CI, −5.1% to −4.9%) (eFigure 3 in Supplement 1). After adjustment, fewer in-person follow-up visits occurred for index visits that were in-person (4.3%) compared with video (14.4%; adjusted difference, 10.1%; 95% CI, 9.9%-10.3%) or telephone (15.1%; adjusted difference, 10.8%; 95% CI, 10.7%-11.0%) visits. Compared with antibiotic prescribing for in-person visits (17.8%), there was less antibiotic prescribing associated with video visits (12.1%; adjusted difference, −5.7%; 95% CI, −5.9% to −5.2%) and telephone visits (10.1%; adjusted difference, −7.7%; 95% CI, −7.9% to −7.5%).

**Figure 2.**
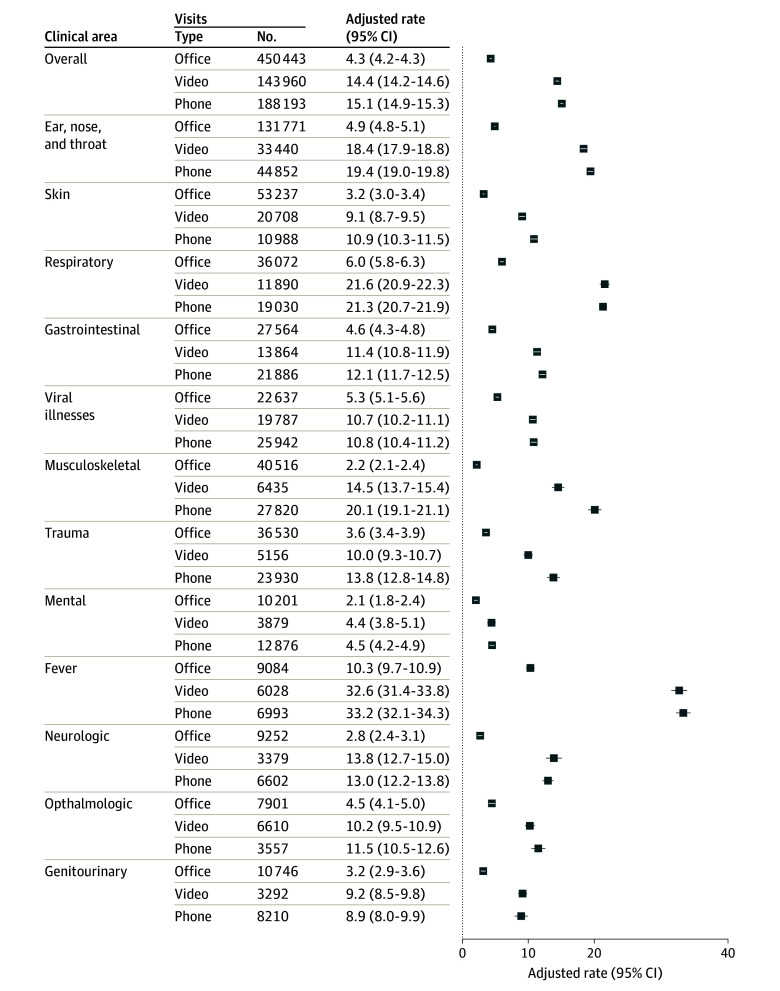
Adjusted Percentage of 7-Day Return Office Visits by Index Visit Type

**Figure 3.**
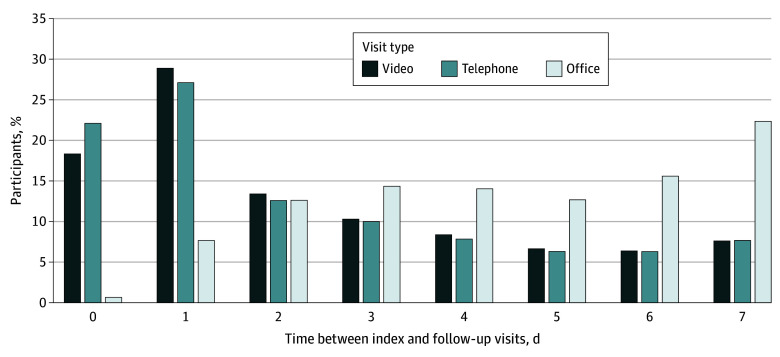
Time Between the Index and Follow-Up Visits for Patients Who Had an In-Person Follow-Up Visit

Adjusted rates of in-person follow-up care after video or telephone appointments were higher across all areas of clinical concern (Figure 2). Most downstream in-person pediatrician follow-up visits following a telephone or video visit were in the first 24 hours of the index visit (49.2% for telephone and 47.3% for video), while most downstream in-person pediatrician visits following an index in-person visit (91.7%) occurred more than 24 hours after the index visit (Figure 3).

**Figure 4.**
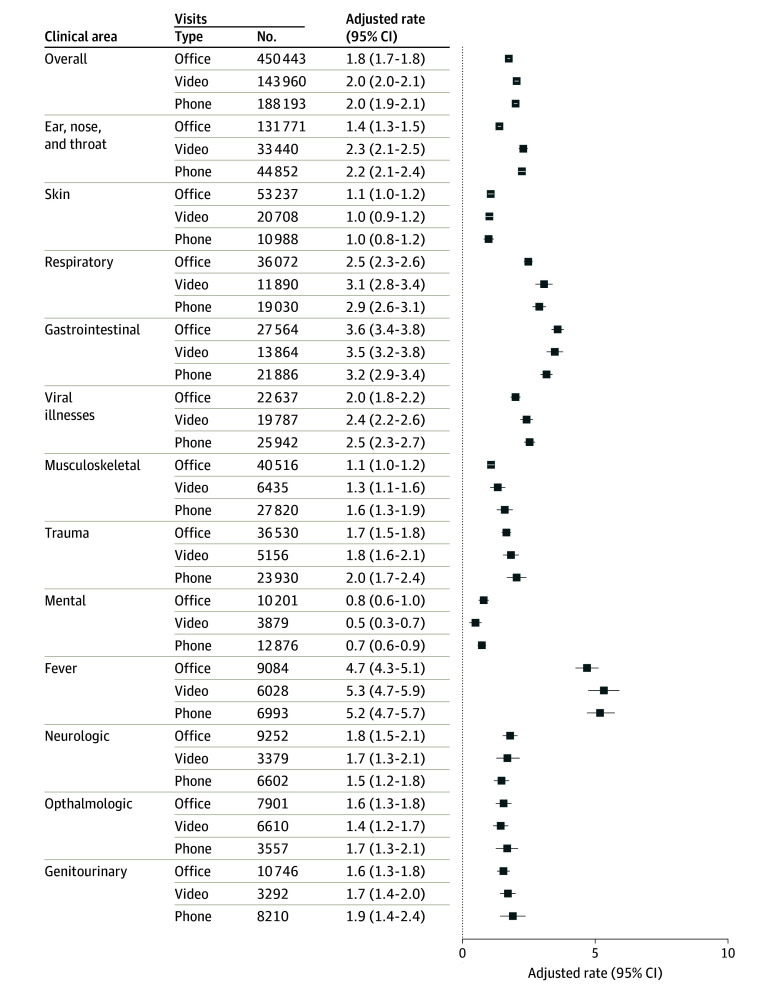
Adjusted Percentage of 7-Day Emergency Department Visits After Index Visit by Visit Type

There were fewer ED visits following an in-person visit (1.75%) compared with after video visits (2.04%; adjusted difference; 0.29%; 95% CI, 0.21%-0.38%) and telephone visits (2.00%; adjusted difference, 0.25%; 95% CI, 0.18%-0.33%) (Figure 4). However, the adjusted percentage of patients hospitalized following an in-person visit (0.14%) was similar to those hospitalized following a video visit (0.12%; adjusted difference, −0.02%; 95% CI −0.04% to 0.00%) or telephone visit (0.08%; adjusted difference, −0.02%; 95% CI, −0.07 to −0.04%).

## Discussion

In this cohort study of a large, integrated health care system in 2022, telephone and video pediatrics primary care visits were associated with less overall physician prescribing and ordering, modest increases in subsequent short-term in-person visits, and small increases in downstream ED encounters, without clinically significant differences in hospitalizations. Telephone or video visits were associated with less prescribing and ordering compared with in-person visits, consistent with previous studies.^11,12,13,20^ Greater physician prescribing observed in previous direct-to-consumer (DTC) telemedicine studies was not observed.^21,22,23,24^ All members of the health system are assigned a primary care physician, and the current study found that a large percentage of scheduled visits were with the patient’s usual pediatrician. Our results may differ from studies of DTC models, as visits in those models occur outside a primary care relationship. A recent study by Wittman et al^25^ noted similar findings and reported lower rates of downstream follow-up visits and antibiotic prescriptions associated with telemedicine visits delivered by primary care physicians compared with DTC practitioners. When stratified by area of clinical concern, telemedicine was associated with lower medication prescribing and imaging and laboratory ordering across all areas of clinical concern except for antibiotic prescribing for ophthalmological concerns.

Slightly more in-person follow-up visits and ED visits occurred after an index video or telephone visit compared with an in-person visit in our study. This result is consistent with telemedicine studies in adults,^12,13^ although it contrasts with the results of 2 small, single-center pediatric telemedicine studies^8,11^ that did not find a statistically significant difference in downstream health care utilization following a telemedicine visit. Reilly et al^8^ did not find an association between telemedicine and increased health care reutilization rates in an urban, academic pediatrics health system during a pandemic period, although their follow-up period was restricted to 72 hours or less. Consistent with our results, Sprecher et al^11^ found that telemedicine was associated with decreased antibiotic prescribing for pediatric telemedicine appointments compared with in-person visits and found no association between telemedicine use and unplanned downstream ED visits. The sample size of 782 596 visits in our study may have provided sufficient power to detect these differences, and additional studies of different health care settings are needed. We found no significant difference in downstream hospitalization rates between visit types. Our results suggest that although telemedicine was associated with more in-person follow up visits, it may not have resulted in excess missed or delayed diagnoses leading to clinical decompensation and subsequent hospitalization.

Notably, 49.2% and 47.3% of the in-person visits scheduled after an index telephone or video visit, respectively, occurred within 1 day of the index visit. This finding appears to support a role for telemedicine in identifying patients who need prompt in-person evaluation to meet their care needs. These promptly scheduled in-person follow-up visits likely reflect in-person visits requested by the pediatrician to collect clinical information only available with a face-to-face interaction, such as vital signs, physical examination findings, or laboratory testing, although we cannot be certain that these visits were driven by pediatricians’ requests or parents’ concerns.^26^ The lack of significant difference in downstream hospitalization suggests that patients who initially made telemedicine visits did not have worse outcomes than those who initially made in-person visits, even if some needed to return for in-person follow-up visits.

### Limitations

These findings should be interpreted considering several limitations. First, in this observational study of outcomes related to visit types, unmeasured confounders may exist. Patients with more comorbidities are likely to require more laboratory and imaging orders, and these patients may have self-triaged to in-person visits. Also, while we adjusted for comorbidities, we were unable to adjust for acuity of the chief concern. Additionally, while we attempted to identify distinct care-seeking episodes by using a 7-day washout period, it is still possible that prescribing, ordering, and downstream visits were unrelated to the index visit. However, this issue may be smaller in a pediatric population, which tends to have fewer chronic comorbidities. Our data were drawn from an all-ages cohort of patients seeking primary care, and Elixhauser score was used to adjust for comorbidities. The Elixhauser score was derived from an adult population and may be insensitive to identify pediatric patients with comorbidities. This study was conducted in a large, integrated health care setting where telemedicine was widely available before the pandemic and the findings may not be generalizable to other settings. Lastly, we are unable to distinguish between downstream in-person visits that were unplanned and visits advised by patients’ pediatricians.

## Conclusions

In this cohort study, primary care pediatric telemedicine was associated with less medication prescribing and laboratory ordering compared with in-person visits across most areas of clinical concern. Based on our results, health care systems and private pediatrics practices seeking to initiate or broaden their pediatric telemedicine programs should expect that primary pediatric care delivered by telephone or video may be associated with modest increases in in-person follow-up visits and slightly higher ED utilization but negligible differences in downstream hospitalizations compared with traditional in-person visits.

## Notes

Supplement 1. **eTable.** Additional covariates included in the model **eFigure 1.** Adjusted percentage of medication prescribing by index visit type **eFigure 2.** Adjusted percentage of laboratory ordering by index visit **eFigure 3.** Adjusted percentage of imaging ordering by index visit type

Supplement 2. Data Sharing Statement
