# Supplementary material for: Health Care Utilization With Telemedicine and In-Person Visits in Pediatric Primary Care
Source: JAMA Health Forum. 2024 Nov 22;5(11):e244156. doi: 10.1001/jamahealthforum.2024.4156 (PMC11584922; doi:10.1001/jamahealthforum.2024.4156)
Supplement: Supplement 1. — eTable. Additional covariates included in the model eFigure 1. Adjusted percentage of medication prescribing by index visit type eFigure 2. Adjusted percentage of laboratory ordering by index visit eFigure 3. Adjusted percentage of imaging ordering by index visit type [file jamahealthforum-e244156-s001.pdf]

# Supplemental Online Content

Casey SD, Huang J, Parry DD, Lieu TA, Reed ME. Health care utilization with telemedicine and in-person visits in pediatric primary care. *JAMA Health Forum.* Published online November 22, 2024. doi:10.1001/jamahealthforum.2024.4156

**eTable.** Additional covariates included in the model

**eFigure 1.** Adjusted percentage of medication prescribing by index visit type

**eFigure 2.** Adjusted percentage of laboratory ordering by index visit

**eFigure 3.** Adjusted percentage of imaging ordering by index visit type

This supplemental material has been provided by the authors to give readers additional information about their work.

© 2024 Casey SD et al. *JAMA Health Forum*.

**eTable 1:** Additional covariates included in models.

| | | All | Office | Video | Telephone |
|---|---|---|---|---|---|
| N | | 782,596 | 450,443 | 143,960 | 188,193 |
| Mobile portal access in prior year | Yes | 3.45 | 3.33 | 3.38 | 3.77 |
| Paid facility parking | Yes | 4.24 | 3.53 | 4.77 | 5.52 |
| ED visit in prior year | Yes | 19.40 | 19.10 | 18.57 | 20.78 |
| Hospitalization in prior year | Yes | 10.82 | 12.97 | 9.51 | 6.67 |
| Appointment booking day | Mon-Thur | 72.65 | 74.31 | 68.33 | 71.96 |
| | Fri | 13.27 | 13.22 | 13.21 | 13.46 |
| | Sat-Sun | 14.08 | 12.47 | 18.46 | 14.59 |
| Visit day | Mon-Thur | 74.78 | 76.95 | 70.41 | 72.93 |
| | Fri-Sun | 25.22 | 23.05 | 29.59 | 27.07 |
| Visit time | Morning | 42.71 | 45.29 | 36.64 | 41.19 |
| | Afternoon | 57.29 | 54.71 | 63.36 | 58.81 |
| Days between booking and visit | 0 day | 41.38 | 36.50 | 48.64 | 47.52 |
| | 1 day | 27.61 | 25.61 | 31.36 | 29.52 |
| | 2-7 days | 22.76 | 25.88 | 17.94 | 19.00 |
| | 8+ days | 8.24 | 12.01 | 2.06 | 3.95 |

© 2024 Casey SD et al. *JAMA Health Forum*.

eFigure 1: Adjusted percentage of medication prescribing by index visit type.

| ClinicalArea | Visit | N | Adjusted Rate (95% CI) |
|---|---|---|---|
| Overall | Office | 450,443 | 39.8 (39.6 to 39.9) |
| | Video | 143,960 | 29.5 (29.3 to 29.7) |
| | Phone | 188,193 | 27.3 (27.1 to 27.5) |
| ENT | Office | 131,771 | 53.5 (53.2 to 53.8) |
| | Video | 33,440 | 27.2 (26.7 to 27.6) |
| | Phone | 44,852 | 26.9 (26.5 to 27.3) |
| Skin | Office | 53,237 | 51.5 (51.0 to 51.9) |
| | Video | 20,708 | 46.4 (45.7 to 47.1) |
| | Phone | 10,988 | 37.9 (37.0 to 38.8) |
| Respiratory | Office | 36072 | 71.0 (70.5 to 71.4) |
| | Video | 11890 | 53.3 (52.4 to 54.2) |
| | Phone | 19030 | 47.0 (46.3 to 47.7) |
| GI | Office | 27,564 | 37.3 (36.7 to 37.8) |
| | Video | 13,864 | 30.3 (29.5 to 31.0) |
| | Phone | 21,886 | 27.1 (26.5 to 27.7) |
| Viral Illnesses | Office | 22,637 | 29.0 (28.4 to 29.6) |
| | Video | 19,787 | 20.0 (19.5 to 20.6) |
| | Phone | 25,942 | 18.1 (17.6 to 18.5) |
| Musculoskeletal | Office | 40,516 | 12.3 (11.9 to 12.7) |
| | Video | 6,435 | 5.7 (5.1 to 6.3) |
| | Phone | 27,820 | 4.1 (3.6 to 4.6) |
| Trauma | Office | 36,530 | 13.2 (12.7 to 13.6) |
| | Video | 5,156 | 9.9 (9.2 to 10.6) |
| | Phone | 23,930 | 6.0 (5.4 to 6.7) |
| Mental | Office | 10,201 | 44.9 (43.8 to 45.9) |
| | Video | 3,879 | 35.4 (33.9 to 37.0) |
| | Phone | 12,876 | 33.1 (32.2 to 34.0) |
| Fever | Office | 9,084 | 31.4 (30.4 to 32.3) |
| | Video | 6,028 | 11.8 (11.0 to 12.7) |
| | Phone | 6,993 | 10.3 (9.6 to 11.0) |
| Neurologic | Office | 9,252 | 23.9 (23.0 to 24.8) |
| | Video | 3,379 | 14.9 (13.7 to 16.1) |
| | Phone | 6,602 | 12.7 (11.9 to 13.5) |
| Eye | Office | 7,901 | 63.2 (62.2 to 64.2) |
| | Video | 6,610 | 57.4 (56.2 to 58.5) |
| | Phone | 3,557 | 57.7 (56.1 to 59.3) |
| GU | Office | 10,746 | 44.1 (43.2 to 45.1) |
| | Video | 3,292 | 28.4 (27.4 to 29.3) |
| | Phone | 8,210 | 31.4 (29.8 to 33.0) |

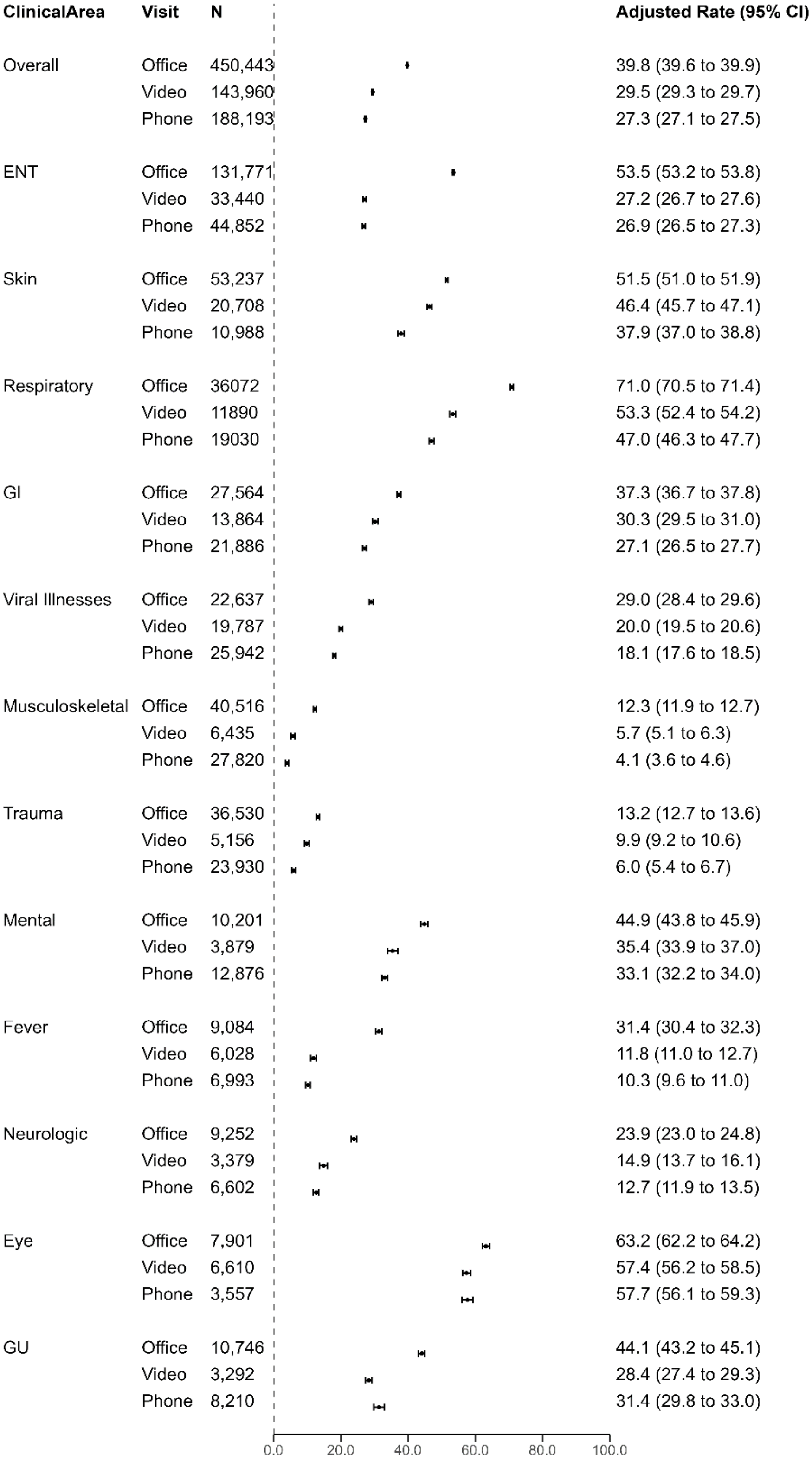


© 2024 Casey SD et al. *JAMA Health Forum*.

eFigure 2: Adjusted percentage of laboratory ordering by index visit.

| ClinicalArea | Visit | N | Adjusted Rate (95% CI) |
|---|---|---|---|
| Overall | Office | 450,443 | 24.6 (24.5 to 24.8) |
| | Video | 143,960 | 7.8 (7.7 to 8.0) |
| | Phone | 188,193 | 8.5 (8.4 to 8.6) |
| ENT | Office | 131,771 | 26.2 (25.9 to 26.4) |
| | Video | 33,440 | 9.5 (9.2 to 9.8) |
| | Phone | 44,852 | 9.4 (9.1 to 9.6) |
| Skin | Office | 53,237 | 13.6 (13.3 to 13.9) |
| | Video | 20,708 | 2.4 (2.2 to 2.6) |
| | Phone | 10,988 | 2.7 (2.4 to 3.0) |
| Respiratory | Office | 36072 | 27.4 (26.9 to 27.9) |
| | Video | 11890 | 5.2 (4.8 to 5.5) |
| | Phone | 19030 | 4.6 (4.3 to 4.9) |
| GI | Office | 27,564 | 32.7 (32.2 to 33.3) |
| | Video | 13,864 | 11.7 (11.1 to 12.2) |
| | Phone | 21,886 | 10.9 (10.5 to 11.3) |
| Viral Illnesses | Office | 22,637 | 39.2 (38.5 to 39.9) |
| | Video | 19,787 | 7.7 (7.3 to 8.0) |
| | Phone | 25,942 | 6.4 (6.1 to 6.7) |
| Musculoskeletal | Office | 40,516 | 10.5 (10.1 to 10.9) |
| | Video | 6,435 | 3.6 (3.1 to 4.0) |
| | Phone | 27,820 | 3.8 (3.3 to 4.2) |
| Trauma | Office | 36,530 | 3.0 (2.8 to 3.2) |
| | Video | 5,156 | 0.6 (0.5 to 0.8) |
| | Phone | 23,930 | 0.6 (0.4 to 0.8) |
| Mental | Office | 10,201 | 12.4 (11.7 to 13.0) |
| | Video | 3,879 | 3.5 (2.9 to 4.1) |
| | Phone | 12,876 | 2.4 (2.2 to 2.7) |
| Fever | Office | 9,084 | 69.3 (68.4 to 70.2) |
| | Video | 6,028 | 19.2 (18.2 to 20.1) |
| | Phone | 6,993 | 17.0 (16.2 to 17.9) |
| Neurologic | Office | 9,252 | 28.2 (27.3 to 29.1) |
| | Video | 3,379 | 10.4 (9.4 to 11.4) |
| | Phone | 6,602 | 8.1 (7.4 to 8.8) |
| Eye | Office | 7,901 | 6.2 (5.6 to 6.7) |
| | Video | 6,610 | 0.9 (0.7 to 1.2) |
| | Phone | 3,557 | 0.7 (0.5 to 1.0) |
| GU | Office | 10,746 | 58.6 (57.7 to 59.5) |
| | Video | 3,292 | 39.3 (38.3 to 40.4) |
| | Phone | 8,210 | 42.1 (40.4 to 43.7) |

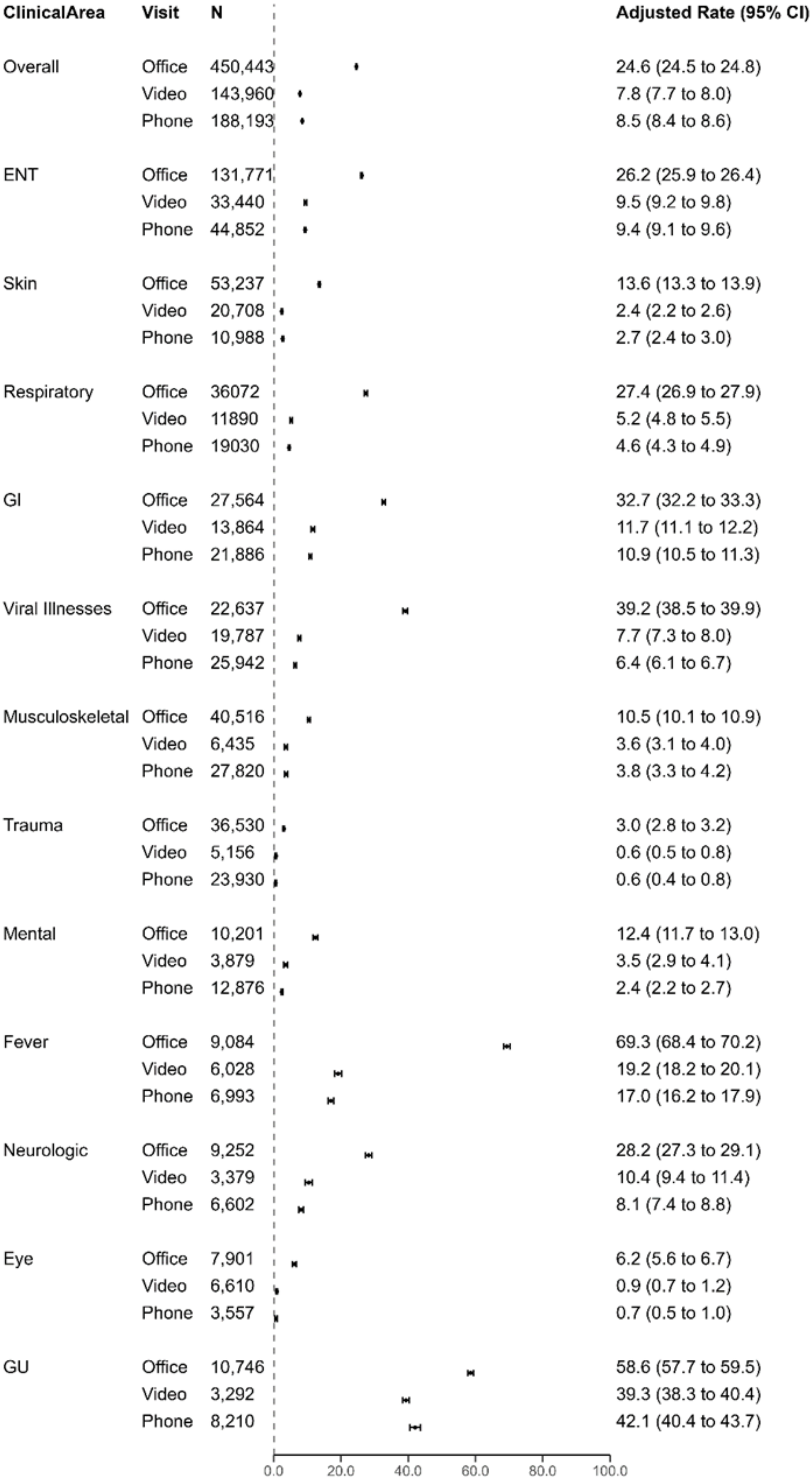


© 2024 Casey SD et al. *JAMA Health Forum*.

eFigure 3: Adjusted percentage of imaging ordering by index visit type.

| ClinicalArea | Visit | N | Adjusted Rate (95% CI) |
|---|---|---|---|
| Overall | Office | 450,443 | 8.5 (8.4 to 8.6) |
| | Video | 143,960 | 4.0 (3.9 to 4.1) |
| | Phone | 188,193 | 3.5 (3.4 to 3.6) |
| ENT | Office | 131,771 | 1.2 (1.1 to 1.2) |
| | Video | 33,440 | 0.4 (0.4 to 0.5) |
| | Phone | 44,852 | 0.4 (0.4 to 0.5) |
| Skin | Office | 53,237 | 2.7 (2.6 to 2.8) |
| | Video | 20,708 | 0.4 (0.3 to 0.5) |
| | Phone | 10,988 | 0.5 (0.4 to 0.7) |
| Respiratory | Office | 36072 | 10.7 (10.4 to 11.0) |
| | Video | 11890 | 4.0 (3.7 to 4.4) |
| | Phone | 19030 | 3.9 (3.6 to 4.2) |
| GI | Office | 27,564 | 12.1 (11.8 to 12.5) |
| | Video | 13,864 | 3.4 (3.1 to 3.7) |
| | Phone | 21,886 | 3.6 (3.4 to 3.9) |
| Viral Illnesses | Office | 22,637 | 2.2 (2.0 to 2.4) |
| | Video | 19,787 | 0.5 (0.4 to 0.6) |
| | Phone | 25,942 | 0.3 (0.2 to 0.4) |
| Musculoskeletal | Office | 40,516 | 51.7 (51.1 to 52.3) |
| | Video | 6,435 | 33.8 (32.7 to 35.0) |
| | Phone | 27,820 | 27.3 (26.2 to 28.4) |
| Trauma | Office | 36,530 | 35.1 (34.5 to 35.7) |
| | Video | 5,156 | 23.4 (22.5 to 24.3) |
| | Phone | 23,930 | 22.3 (21.2 to 23.4) |
| Mental | Office | 10,201 | 1.4 (1.1 to 1.6) |
| | Video | 3,879 | 0.4 (0.2 to 0.6) |
| | Phone | 12,876 | 0.2 (0.1 to 0.3) |
| Fever | Office | 9,084 | 7.8 (7.2 to 8.3) |
| | Video | 6,028 | 1.7 (1.3 to 2.0) |
| | Phone | 6,993 | 1.7 (1.4 to 2.0) |
| Neurologic | Office | 9,252 | 5.5 (5.0 to 6.0) |
| | Video | 3,379 | 1.7 (1.2 to 2.1) |
| | Phone | 6,602 | 1.5 (1.2 to 1.8) |
| Eye | Office | 7,901 | 0.5 (0.3 to 0.6) |
| | Video | 6,610 | 0.1 (0.0 to 0.2) |
| | Phone | 3,557 | 0.3 (0.1 to 0.5) |
| GU | Office | 10,746 | 5.2 (4.8 to 5.6) |
| | Video | 3,292 | 2.3 (2.0 to 2.6) |
| | Phone | 8,210 | 2.2 (1.7 to 2.7) |

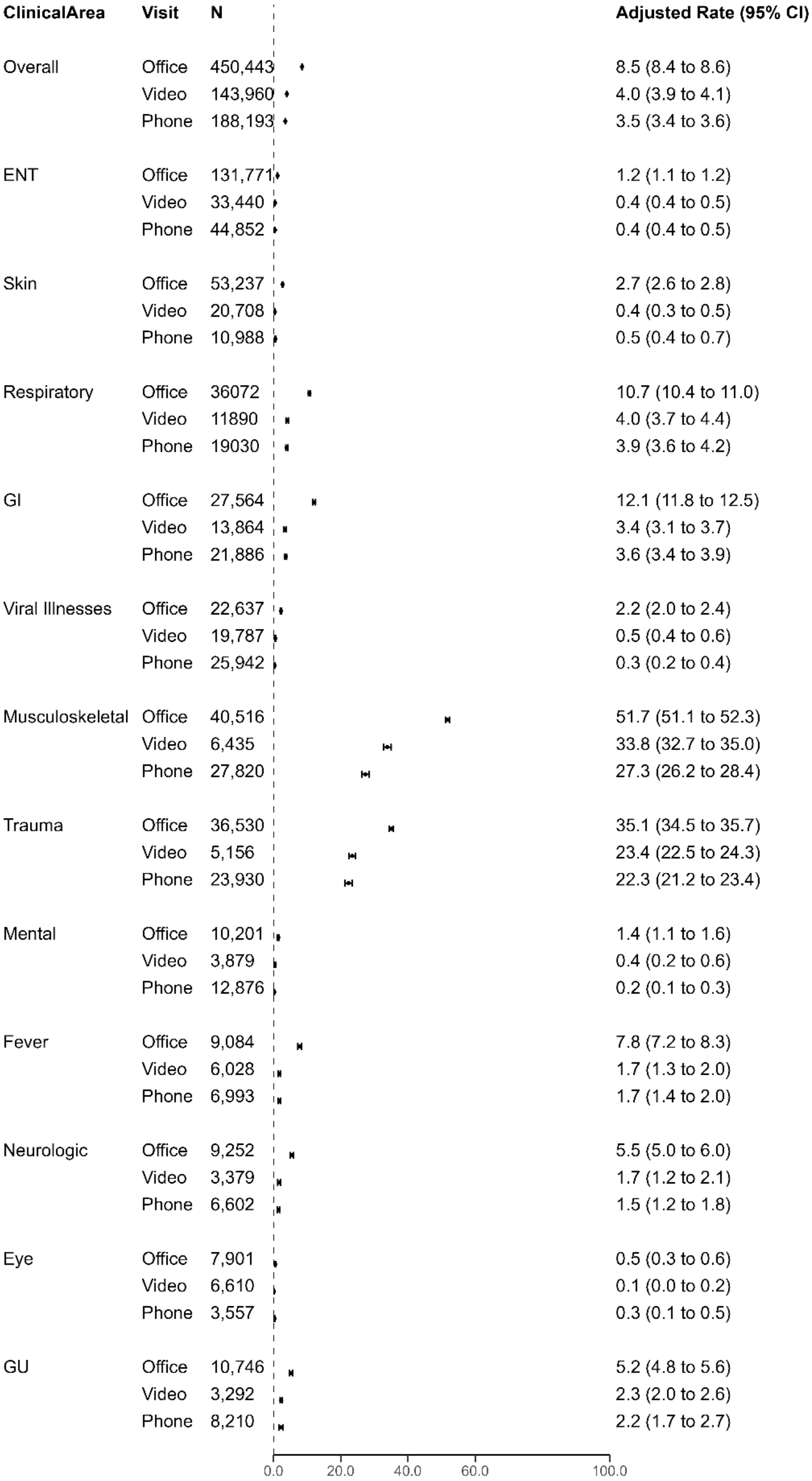


© 2024 Casey SD et al. *JAMA Health Forum*.
