# Supplementary material for: Health Care Utilization With Telemedicine and In-Person Visits in Pediatric Primary Care
Source: JAMA Health Forum. 2024 Nov 22;5(11):e244156. doi: 10.1001/jamahealthforum.2024.4156 (PMC11584922; doi:10.1001/jamahealthforum.2024.4156)
Supplement: Supplement 2. — Data Sharing Statement [file jamahealthforum-e244156-s002.pdf]

Data Sharing Statement

Casey. Health Care Utilization With Telemedicine and In-Person Visits in Pediatric Primary Care. *JAMA Health Forum*. Published November 22, 2024.
doi:10.1001/jamahealthforum.2024.4156

Data
Data available: Yes
Data types: Other (please specify)
Additional Information: Deidentified data may be available upon request but will necessitate review from our legal department depending on the data requested.
How to access data: Deidentified data may be available upon request but will necessitate review from our legal department depending on the data requested.
When available: With publication

Supporting Documents
Document types: Statistical/analytic code
How to access documents: Deidentified data may be available upon request but will necessitate review from our legal department depending on the data requested.
When available: With publication

Additional Information
Who can access the data: Deidentified data may be available upon request but will necessitate review from our legal department depending on the data requested.
Types of analyses: Deidentified data may be available upon request but will necessitate review from our legal department depending on the data requested.
Mechanisms of data availability: Deidentified data may be available upon request but will necessitate review from our legal department depending on the data requested.
Any additional restrictions: Deidentified data may be available upon request but will necessitate review from our legal department depending on the data requested.
